# A world report on the transformation needed in mental health care

**DOI:** 10.2471/BLT.22.289123

**Published:** 2022-10-01

**Authors:** Dévora Kestel, Sian Lewis, Melvyn Freeman, Dan Chisholm, Olivia Gascoigne Siegl, Mark van Ommeren

**Affiliations:** a Department of Mental Health and Substance Use, World Health Organization, Avenue Appia 20, 1211 Geneva 27, Switzerland.; b London, England.; c Johannesburg, South Africa.; d Birmingham, England.

The coronavirus disease 2019 (COVID-19) pandemic, along with other global threats such as socioeconomic inequalities, conflict and climate change, has increased the need to address mental health.^1^ Interest in mental health, already on the rise before the pandemic, is currently at an all-time high, with mental health increasingly figuring on public health, human rights, development and peacebuilding agendas.^2^ However, countries are not on course to reach the targets of the World Health Organization’s (WHO) *Comprehensive mental health action plan 2013–2030*^3^ nor the sustainable development goals’ target for suicide mortality.

In June 2022, WHO released the *World mental health report: transforming mental health for all*.^4^ This report sets out to inspire and inform all stakeholders about transforming actions needed for mental health. Accompanied by narratives of lived experiences of mental health conditions, the report presents the public health, human rights and socioeconomic development arguments for investment in mental health. The report also describes the routes to transformation, showcasing strategies proven to work, including in low-income countries.

Mental health is an integral part of our general health and well-being and a basic human right. A diverse set of interacting individual, family, community and structural factors protects or undermines our mental health. Although most people are resilient, people who are exposed to adverse circumstances – including poverty, violence and inequality – are at higher risk of mental health conditions. Because the factors determining mental health are multisectoral, interventions to promote and protect mental health must occur across sectors.

The report presents the latest data on mental health. Globally, suicide – a major cause of death in young people – accounts for more than one in every 100 deaths, and mental disorders continue to be the leading cause of years lived with disability. In 2019, an estimated 970 million people were living with a mental disorder. Between 2000 and 2019, the estimated point prevalence of mental disorders remained stable, at 13% of the world’s population. Depression and anxiety are the most prevalent mental disorders, and these are more common among females.^4^ During the first year of the pandemic, the prevalence of anxiety and depressive disorders increased by an estimated 25%.^1^ Yet in terms of service coverage across the world, only about one in three people with serious conditions such as psychosis and depression receive mental health services.^4^

The report describes three pathways to accelerate progress in implementing the action plan. The first involves deepening value and commitment to mental health, with a general call to understand and appreciate mental health and invest time and effort into taking care of one’s own mental health as well as supporting others. This pathway is also about including people with mental health conditions in all aspects of society and decision-making, and to give mental health the same value and priority as physical health. Ultimately, deepening value and commitment to mental health is about stepping up investments in mental health with financial and human resources, and evidence-based policies and practice that uphold human rights.

The second pathway to transformation is to reshape the social and natural environments that can protect or adversely affect mental health, by prioritizing action to advance prevention of all forms of violence, coercion, abuse, neglect, discrimination, bullying and harassment, including of people with mental health conditions; enable nurturing care in early life and social and emotional learning programmes in schools; secure decent working conditions at work; and protect and improve our natural environments.

The third pathway to transformation is to strengthen mental health care to ensure everyone can access the care they need. Strengthening mental health care means delivering person-centred, human rights-based, recovery-oriented mental health care through community-based networks of interconnected services in secondary and primary health-care settings, complemented with support by community providers (such as community workers and peers). Importantly, strengthening mental health care means moving away from custodial care in psychiatric hospitals as community-based services become available, and diversifying and scaling up care options for depression and anxiety, including through task-sharing and digital technologies. Strengthening mental health care also means making quality mental health care accessible and affordable for all, including through meaningful integration of mental health care in universal health coverage.

Many countries do not have the resources to implement all actions described in this report. But every country has opportunities to make significant progress towards improving the mental health of its people.
